# ﻿Metabarcoding of insect-associated fungal communities: a comparison of internal transcribed spacer (ITS) and large-subunit (LSU) rRNA markers

**DOI:** 10.3897/mycokeys.88.77106

**Published:** 2022-03-08

**Authors:** Angelina Ceballos-Escalera, John Richards, Maria Belen Arias, Daegan J. G. Inward, Alfried P. Vogler

**Affiliations:** 1 Department of Life Sciences, Natural History Museum, Cromwell Road, London SW7 5BD, UK Natural History Museum London United Kingdom; 2 Department of Life Sciences, Imperial College London, Silwood Park Campus, Ascot SL5 7PY, UK Imperial College London Ascot United Kingdom; 3 Forest Research, Alice Holt Research Station, Farnham, Surrey, GU10 4LH, UK University of Essex Colchester United Kingdom; 4 School of Life Sciences, University of Essex, Colchester Campus, CO4 3SQ, UK Forest Research, Alice Holt Research Station Farnham United Kingdom

**Keywords:** clustering, fungi, ITS, LSU, metabarcoding, pathogens, phylogeny, Scolytinae

## Abstract

Full taxonomic characterisation of fungal communities is necessary for establishing ecological associations and early detection of pathogens and invasive species. Complex communities of fungi are regularly characterised by metabarcoding using the Internal Transcribed Spacer (ITS) and the Large-Subunit (LSU) gene of the rRNA locus, but reliance on a single short sequence fragment limits the confidence of identification. Here we link metabarcoding from the ITS2 and LSU D1-D2 regions to characterise fungal communities associated with bark beetles (Scolytinae), the likely vectors of several tree pathogens. Both markers revealed similar patterns of overall species richness and response to key variables (beetle species, forest type), but identification against the respective reference databases using various taxonomic classifiers revealed poor resolution towards lower taxonomic levels, especially the species level. Thus, Operational Taxonomic Units (OTUs) could not be linked via taxonomic classifiers across ITS and LSU fragments. However, using phylogenetic trees (focused on the epidemiologically important Sordariomycetes) we placed OTUs obtained with either marker relative to reference sequences of the entire rRNA cistron that includes both loci and demonstrated the largely similar phylogenetic distribution of ITS and LSU-derived OTUs. Sensitivity analysis of congruence in both markers suggested the biologically most defensible threshold values for OTU delimitation in Sordariomycetes to be 98% for ITS2 and 99% for LSU D1-D2. Studies of fungal communities using the canonical ITS barcode require corroboration across additional loci. Phylogenetic analysis of OTU sequences aligned to the full rRNA cistron shows higher success rate and greater accuracy of species identification compared to probabilistic taxonomic classifiers.

## ﻿Introduction

Fungal communities associated with insects have been widely studied to disentangle the ecological roles and specificities of these interactions (Ganter 2006; Raman et al. 2012; Li et al. 2016; Malacrinò et al. 2017; Jacobsen et al. 2018). For these studies to succeed, accurate and reliable fungal identifications are essential. However, identifications of fungi are challenging due to their cryptic morphology and incomplete taxonomy, with only 3–8% of fungal species described so far (Hibbett et al. 2016; Kandawatte Wedaralalage et al. 2020). Conventional studies of fungal communities have been conducted by isolating and culturing the fungi associated with insect specimens (Batra 1963), but this overlooked many unculturable species. High-throughput DNA sequencing has provided an alternative methodology by amplifying and sequencing short ‘barcodes’ from mixed communities (metabarcoding) (Yu et al. 2012). Metabarcoding is now widely applied in characterising the species composition and diversity of fungal communities associated with insects. In the specific case of fungal communities associated with bark beetles, metabarcoding usually detects dozens of species of fungi isolated from a single insect specimen (Bálint et al. 2014; Miller et al. 2016, 2019, Malacrinò et al. 2017; Johnson et al. 2018; Hulcr et al. 2020).

There is broad agreement that the internal transcribed spacer (ITS) of the nuclear rRNA gene cluster should be the standard DNA barcode in fungi (Schoch et al. 2012). Its utility in metabarcoding is now equally well established, and extensive reference databases and universal primer combinations are in wide use (Porras-Alfaro et al. 2014; Tedersoo et al. 2015b). However, various challenges remain for accurate characterisation of communities. PCR amplification biases may skew species recovery (Bellemain et al. 2010; Harrington et al. 2011; Dreaden et al. 2014; Tedersoo et al. 2015b; Li et al. 2020). For example, the ITS marker may not detect key pathogen species in the Ophiostomatales (Skelton et al. 2019; Hulcr et al. 2020). In addition, the recovered short sequence fragments have limited power for phylogenetic placement (Vrålstad 2011; Porras-Alfaro et al. 2014), exacerbated by the incompleteness of the reference databases (Porras-Alfaro et al. 2014; Tedersoo et al. 2015a; Miller et al. 2016; Agerbo Rasmussen et al. 2020). In response to these challenges, several fungal phylogenetic and barcoding studies have used a combination of ITS and partial large and small subunit (LSU and SSU) rRNA genes, as well as other markers such as RPB2 and TEF1α (Lutzoni et al. 2004; Zhang et al. 2006; Stielow et al. 2015). Extensive curated reference sets and analysis tools like SILVA and RDP (Ribosomal Database Project) have been built specifically for SSU and LSU genes (Wang et al. 2007; Quast et al. 2012).

In practice, both the ITS and LSU/SSU markers exhibit particularities whose benefits and drawbacks depend on the aim and scope of a study (Porras-Alfaro et al. 2014). The LSU/SSU genes are less variable than the ITS intergenic regions, which favours alignment and tree-based analyses, but their low rate of molecular evolution reduces the taxonomic resolution at the species-level. In turn, ITS provides better species resolution due to its higher substitution rate but, as a non-coding RNA, the ITS region is prone to insertion/deletions, which causes difficulties with alignment and phylogenetic analysis (Vrålstad 2011; Porras-Alfaro et al. 2014). In addition, the higher substitution rate in ITS leads to intragenomic variation of the tandem repeat units, given the slow homogenisation among the various copies. However, in fungi this intraspecific and intragenomic variation is still poorly documented, and it may also affect the LSU/SSU coding regions (Lücking et al. 2020). The differences in evolutionary rates and in levels of intra-genomic variation have implications for the way the raw reads are processed in ecological and taxonomic studies. In metabarcoding, sequence reads are usually clustered into Operational Taxonomic Units (OTU) to circumscribe and identify fungal species (Hyde et al. 2013; Kõljalg et al. 2013; Hibbett et al. 2016; Kandawatte Wedaralalage et al. 2020; Lücking et al. 2020). However, if the two regions evolve at different rates, this may affect the optimal threshold values of clustering in establishing the species level entities, and equally may change the interpretation of quality filtered reads, the so-called Amplified Sequence Variants (ASVs) (Callahan et al. 2017), to represent the haplotypes of individuals.

The problem of marker choice and the comparability of metabarcoding studies using either type could be alleviated if both regions were sequenced for the same specimens. Whilst this is a powerful approach for cultured isolates (Vu et al. 2019), it is not possible to link ITS and SSU/LSU amplicons in the metabarcoding mixtures. A recent study attempted to perform metabarcoding of longer amplicons covering both markers with long-read technology, which is ultimately the way forward, but laboratory and bioinformatic procedures currently developed for short fragments could not be applied easily (Furneaux et al. 2021). Thus, short fragments of either marker remain the focus of metabarcoding for the immediate future, which leaves the question about the consequences of marker choice for the conclusions from such studies. To date the issue of ITS vs. LSU comparability has mainly been addressed by conducting amplification of both markers from the same mixture, both in mock (Bakker 2018; Egan et al. 2018; Frau et al. 2019) and natural communities (Parada et al. 2016; Jusino et al. 2019; Li et al. 2020). When applied to the study of ecological patterns these studies have found no major effect of the marker choice (Tedersoo et al. 2015b; George et al. 2019; Nilsson et al. 2019; Furneaux et al. 2021). However, these studies generally have applied a coarse-grain approach of higher-level taxonomic analysis, rather than the species level, where the effects of using different reference databases and different clustering methods may be more pronounced.

Here, we address the problem of identification and unification of information derived from both markers using phylogenetic approaches. Metabarcodes obtained from a given community, as those associated with a single insect, should be composed of the same lineages, and thus occupy the same positions in a phylogenetic tree. Generating trees independently for ITS and LSU does not overcome the problem of associating the sequences from both amplicons, and hence the aim here is to integrate these sequences in the same tree. This may be achieved based on a scaffold of well-identified reference sequences covering the entire rRNA cluster, including ITS and LSU, to which the non-overlapping sequences for each marker are added for a joint tree search. If both markers represent the same fungal community, the corresponding ITS and LSU sequences should appear in a similar place in the tree, relative to a given reference sequence spanning both regions. Besides the greater precision of the phylogenetic position, the use of both barcodes in a single analysis also overcomes the problem of using different reference sets in the prevailing databases for ITS and rRNA markers.

We test this approach for fungal communities associated with bark beetles (Coleoptera: Scolytinae). These insects breed in living or dead trees and form close associations with fungi, which are important for access to nutrients from wood that cannot be utilised directly by the beetles themselves (Batra 1963). Fungal communities associated with these beetles are highly diverse and form symbioses of varying strength and specificity, and may involve the active transport of fungal hyphae or spores in specifically adapted pockets of the beetles’ exoskeleton, the mycangia (Six 2020). The beetle-fungus complex can cause enormous damage to forest ecosystems, e.g., resulting in the demise of chestnuts in North America and elms across the Northern Hemisphere, or the recent large-scale decline of conifer forests in Central Europe and North America, which usually involve fungi from the ascomycete orders Ophiostomatales, Microascales and Hypocreales (Class Sordariomycetes) (Ploetz et al. 2013). Metabarcoding now provides a powerful tool for detailed studies of these complex communities, but the results may be influenced by the choice of barcode markers and various experimental problems in using short sequences from mixed amplicons, such as primer bias and co-amplification of paralogues. We used individuals from four bark beetle species obtained from three forest types to characterise the associated fungal communities, conducting a comparison of the two markers with regard to: (1) broad ecological trends of fungal associations taking a whole-community approach, and (2) species identifications against existing ITS and LSU fungal reference databases, using various taxonomic classifiers and explicit phylogenetic methods. The side-by-side comparison addresses the power of either marker to infer critical parameters of fungal community metabarcoding, such as the number and taxonomic identity of OTUs, their ecological associations, and inference of whole-community diversity and turnover. The phylogenetic approach also can improve upon the taxonomic placement of OTUs conducted with probabilistic classifiers.

## ﻿Materials and methods

### ﻿Samples used and laboratory procedures

Sequence data were generated from 20 specimens per species for four species (*Xylosandrusgermanus*, *Xyleborinussaxesenii*, *Gnathotrichusmateriarius*, and *Tomicuspiniperda*), for a total of 80 specimens (Table 1). Only the latter is a xylophagous ‘bark beetle’ in the strict sense, while the three others are considered mycelia feeding (xylomycetophagous) ‘ambrosia beetles’ that rely on active transport of fungi indicated by the presence of mycangia (see Six 2020). Specimens were collected by Forest Research UK (Alice Holt, Hampshire, UK, see https://www.forestresearch.gov.uk/) during 2013–2015 in the New Forest National Park (50°50'52.08"N, 1°35'33.51"W), Hampshire, UK, using Lindgren multiple-funnel traps (Lindgren 1983) (Phero Tech). These traps were placed in oak, spruce and pine forests and were baited with lures (100% ethanol, plus α-pinene) (Inward 2019). Propylene glycol (65%) was used as the preservation fluid at the bottom of the traps. Specimens were morphologically identified and selected at random to obtain the same number of specimens per beetle species and forest type.

**Table 1.** Beetle species included in the study and relevant life history information.

Forest type	Beetle species	Status	Adapted structures	Feeding mode
Spruce, oak	*Xylosandrusgermanus*	Introduced	Mesonotal mycangia	Xylomycetophagous
Spruce, oak	*Xyleborinussaxesenii*	Native	Elytral mycangia	Xylomycetophagous
Pine, spruce	*Gnathotrichusmateriarius*	Introduced	Tubular opening near precoxae	Xylomycetophagous
Pine, spruce	*Tomicuspiniperda*	Native	No known mycangia	Xylophagous

In the laboratory, the specimens were rinsed with pure water to remove loosely adhering fungal tissue, and thoroughly macerated individually to ensure that all fungi associated with the specimens were released. DNA was extracted using the DNeasy Blood and Tissue spin column extraction kit (Qiagen, Valencia, CA, USA). Individual DNA extracts were first tested for correct beetle species identification using the COI barcode marker, which was amplified for a 418 bp fragment and sequenced on Illumina HiSeq following methods of Arribas et al. (2016). In all cases the most abundant read, as determined with the NAPselect script (Creedy et al. 2019), had an exact match to existing reference sequences of the respective species, confirming the morphological identification.

The DNA extracts were then used for fungal metabarcoding of the ITS2 region with primers ITS86F (5′-GTGAATCATCGAATCTTTGAA-3′) (Op De Beeck et al. 2014)/ ITS4 (5′-TCCTCCGCTTATTGATATGC-3′) (White et al. 1990) and LSU using primers LR0R (5′-ACCCGCTGAACTTAAGC-3 (Vilgalys and Hester 1990)/ JH-LSU-369rc (5′-CTTCCCTTTCAACAATTTCAC-3′) (Li et al. 2016) targeting the D1-D2 region at the 5’ end of the LSU gene immediately downstream of the ITS2 region. Both markers were amplified from each beetle DNA extraction in separate reactions. Unique six-nucleotide indices added to each primer pair were used to distinguish the libraries. PCRs were pooled from three replicates conducted under slightly different annealing temperatures (54 °C, 55 °C and 56 °C) to accommodate differences in optimal amplification conditions of the fungal species (Schmidt et al. 2013), and blank PCR reactions were used as negative control. Successful PCR amplicons were purified using the AMPure XP magnetic beads (Beckman Coulter). Amplicons were indexed using a secondary PCR with Nextera XT DNA Library Preparation Kit (Illumina Inc.) and sequenced on an Illumina HiSeq 2500 platform to generate 2 × 300 bp paired-end reads.

### ﻿Bioinformatics

Raw reads were demultiplexed, primer sequences trimmed, and singleton reads removed with Cutadapt v. 2.10 (Martin 2011). Read quality was evaluated using FastQC v. 0.11.9 (Andrews et al. 2010). The raw reads generated for these analyses are available as Bio-Project PRJNA727174 (Sequence Read Archives) in the BioSample Submission Portal (Barrett et al. 2012).

Forward and reverse reads were merged and quality filtered (Phred score ≥ 30) using PEAR v. 0.9.8 (Zhang et al. 2014), while un-merged reads were discarded. After merging, the average read length was 252 bp for ITS2 and 357 bp for LSU D1-D2. Subsequent steps were carried out using VSEARCH v. 2.15.0 (Rognes et al. 2016) using the following commands. A further quality test was conducted using the --fastx_filter command and --fastq_maxee 1.0. After dereplication (--derep_fulllength), assemblies were denoised (--cluster_unoise --minsize 4 --unoise_alpha 2) and length filtered for a range of 100 to 500 bp (--fastx_filter) and all singletons removed. Chimera filtering was performed with --uchime3_denovo and reads were then clustered into Operational Taxonomic Units (OTUs) at various similarity thresholds (97%, 98%, 99%) using the --cluster_size command. The average length of the OTU representative sequences was 270 bp for ITS2 and 347 bp for LSU D1-D2 (Suppl. material 1: Fig. S1). Reads were then mapped to the 97% OTU clusters, outputting an OTU table of read abundances suitable for the ecological analysis.

### ﻿OTU identification and classification

Fungal OTUs were classified following three widely used methods for species identification. The Ribosomal Database Project (RDP) Bayesian Classifier (Wang et al. 2007) was used for fungal identification employing the Warcup fungal ITS (v. 2, release March 2018) and UNITE (accessed on February 2020) training sets (Deshpande et al. 2016; Edgar 2018). In addition, OTUs were processed through the Protax-fungi pipeline (Abarenkov et al. 2018), implemented in the PlutoF platform (Abarenkov et al. 2010) and based on the UNITE fungal database (accessed February 2020). Protax-fungi hierarchically assigns the OTU identities from the root node of the taxonomy through to the species (Nilsson et al. 2019). It has not been implemented for LSU, and thus was applied to the ITS data only. A third classifier, IDTAXA, employs machine learning to reduce over-classification errors to obtain a higher accuracy (Murali et al. 2018). Taxonomic assignment was carried out separately on class, order, genus, and species level. A minimum threshold of 70% confidence for at least one of the classifiers was set, below which the OTUs were considered as “unclassified”, together with other sequences that were identified with high confidence against database entries labelled as “unclassified”, “unidentified” or “*incertae sedis*”. Then, for the remaining identifications, the confidence values were averaged (average of three values for ITS2 and two for LSU D1-D2 data). When identifications disagreed among the classifiers, the one with the highest confidence value was selected, although this could give preference to over-confident classifiers, i.e., RDP (2018). Taxonomic composition of samples was presented as the number of OTUs assigned to a given taxonomic level in a barplot created with *ggplot2* in Rstudio (Wickham 2016) and was used for the ecological analysis. In addition, in a more detailed study of OTU assignments in the ecologically important class Sordariomycetes, the identification provided by the three classifiers was compared to their position in a phylogenetic tree (see below).

The Sordariomycetes subset was also used to test the effect of variable sequence similarity thresholds on the classification, by generating OTUs under clustering at 97%, 98%, and 99% similarity and comparing the taxonomic assignments, using the RDP classifier (Warcup 2 and Fungal 11 training sets for ITS2 and LSU D1-D2, respectively) (Deshpande et al. 2016). All OTUs with a confidence of assignment > 70% to class Sordariomycetes were retained. Order-level assignments (the Sordariomycetes are split into 28 orders) with a confidence > 50% were taxonomised, while all others were kept as “unclassified Sordariomycetes”. To assess the effects of differing clustering thresholds on downstream taxonomic assignment, OTUs at each clustering threshold were also closed-reference clustered (i.e., only retaining sequences with hits in the reference set) against the composite LSU/ITS reference sequences used to construct the tree (Edgar 2010; Rognes et al. 2016).

### ﻿Alignment and tree building in Sordariomycetes

Reference sequences for the class Sordariomycetes were downloaded from Genbank, querying the database for various permutations of the gene names for the rRNA cluster composed of SSU, LSU and ITS, separately for each target fungal order. Only sequences that were complete for at least 2/3 of the rRNA operon were chosen (full list of accessions in Suppl. material 5: Table S1). 80% of species in this reference set were complete for all three regions. ITS2 reference sequences were processed through ITSx to eliminate redundancy in the concatenated alignment (Bengtsson-Palme et al. 2013).The subsequent steps were carried out separately for each OTU set at 97%, 98% and 99% clustering thresholds. The reference sequences and OTU representative sequences were aligned using MUSCLE (Edgar 2004) under default settings and the aligned matrices were concatenated. The concatenated three-region alignment (SSU, LSU, ITS1-2) was then inspected in Mesquite (Maddison and Maddison 2021) and Geneious Prime (v. 2020.0.4) and problematic accession sequences were removed. This alignment is available on TreeBase (www.treebase.org accession number S28904). The alignment was then partitioned for each marker region, and the best model for each partition was selected according to BIC values. Model testing, tree building, and ultrafast bootstrap approximation (n = 1000) were performed in IQ-Tree2 (Chernomor et al. 2016). Tree visualisation was improved using iTOL v. 6.5 (Letunic and Bork 2007).

### ﻿Phylogenetic diversity metrics

Phylogenetic distribution of ITS2 and LSU D1-D2 copies was assessed by metrics of clustering and over-dispersion originally developed for community ecology (Webb et al. 2008). In the ideal case of capturing the same species with both markers, copies of ITS2 and LSU D1-D2 corresponding to the same species should be in close vicinity on the tree, i.e., the copies of each marker should be ‘over-dispersed’ (more dispersed than a random phylogenetic structure). Deviations from this pattern can be assessed with the metrics calculating the Mean Pairwise Distances (MPD) and Mean Nearest Taxon Distances (MNTD) of each set (ITS2 and LSU D1-D2). We report standardised values as the net relatedness index (NRI) and nearest taxon index (NTI) relative to null models of randomly distributed communities. Positive NRI and NTI scores indicate phylogenetic clustering, negative values indicate phylogenetic over-dispersion, while random phylogenetic structure results in values not significantly different from zero (Webb et al. 2008). Calculations were performed with the *R* packages *picante*, *ape*, and *phylomeasures* (Webb et al. 2008; Tsirogiannis and Sandel 2016; Paradis and Schliep 2019).

### ﻿Assessment of species richness and community composition

Community ecological analyses were carried out on samples rarefied to 1000 reads, which was sufficient for generating largely complete OTU sets as judged by species accumulation curves (Suppl. material 2: Fig. S2). Species accumulation curves were built with the *specaccum* function of the *vegan* package (Oksanen et al. 2013). An OTU table and species classification was generated for fungal communities separately from ITS2 and LSU D1-D2 sequencing, after singletons and doubletons were removed. For the OTU table, the 97% threshold was selected because it is the most generally applied in fungal studies (Nilsson et al. 2008). Fungal OTU richness among samples was assessed with a Generalised Linear Model (GLM) built with the *lme4* package (Bates et al. 2015), with fungal OTU richness as a response variable and beetle species and forest type as dependent variables. The Negative Binomial model was chosen, as it is suitable for overdispersed data. A post hoc pairwise comparison (Tukey HSD test at the 95% significance level) was carried out to compare the means among the distinct factors.

The Jaccard index was used to calculate beta-diversity between sample pairs based on OTU presence-absence data (richness) (*betapart* R package; Baselga and Orme 2012). The variation was visualised using Nonmetric Multidimensional Scaling (NMDS) (*metaMDS* function of the vegan package; Oksanen et al. 2013). To evaluate the stringency of association of fungal OTUs with tree species and beetle hosts for each assembly, a multilevel pattern analysis was carried out by calculating Pearson’s phi coefficient of association (“p.g”) (Chytrý et al. 2002) between sample pairs, correcting this index to account for the differences in specimen numbers among the compared groups (function *multipatt* of the *indicspecies R* package; (De Cáceres et al. 2011). OTUs for which the association values were significant were displayed as a heatmap (*aheatmap* function, NMF *R* package (Gaujoux and Seoighe 2010).

## ﻿Results

### ﻿Composition of fungal communities from ITS and LSU markers

**Figure 1.**
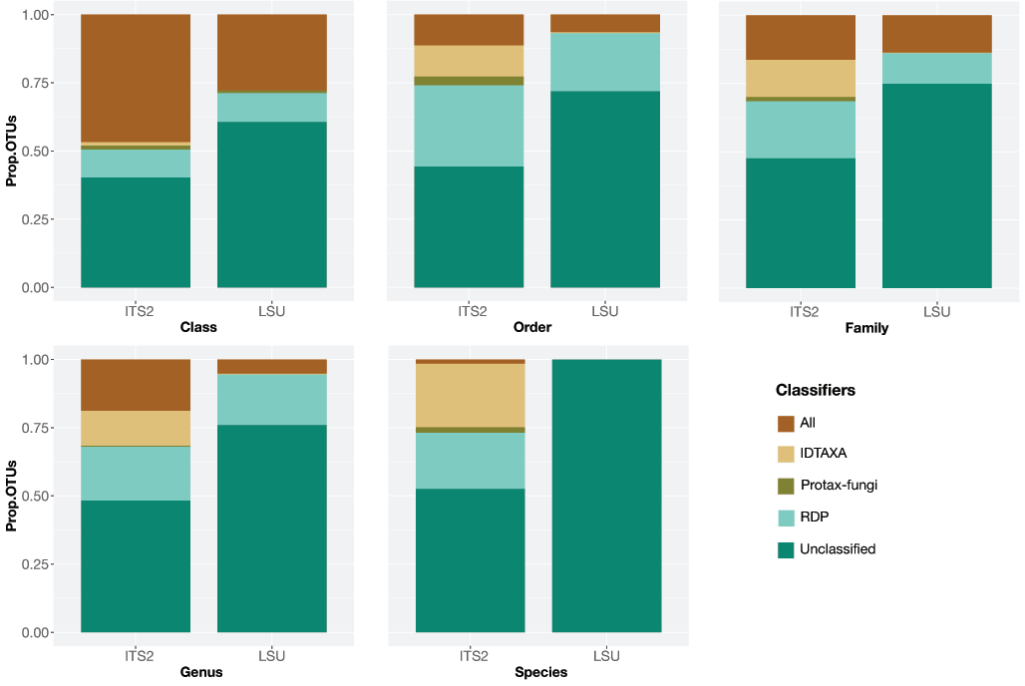
The proportion of fungi classified with IDTAXA, Protax-fungi and RDP from class to species level. “All” refers to the proportion of OTUs for which the three classifiers agreed in their classification.

Sequencing of 80 libraries produced 2,436,075 quality-filtered, merged reads for ITS2 and 1,742,119 reads for LSU D1-D2, which resulted in 1157 OTUs from ITS2 and 548 OTUs from LSU D1-D2 after bioinformatics filtering and clustering at 97% threshold (1546 and 632 OTUs if singleton and doubleton reads were retained and without applying rarefaction on each library). Identifications of OTUs at ≥ 70% confidence level obtained with IDTAXA, Protax-fungi and RDP were higher for ITS2 than for LSU D1-D2 at all hierarchical levels from class to order, family, genus and species level (Fig. 1). However, the fraction of OTUs identified by one or multiple identifiers never exceeded 61.5% for ITS2 and 41.5% for LSU D1-D2 of the total OTUs. Identifications dropped consistently from class to species level, and with each hierarchical level an increasing proportion of identifications was due to a single classifier only, indicating the growing uncertainty of taxonomic assignments. A classification at species-level was generally not possible for LSU because of the limitations of the databases, which generally provide a taxonomy string to genus level only and nearly 100% of the OTUs remained unidentified at this level. Nearly 50% of the ITS2 OTUs were identified to species level but in almost all cases only a single classifier produced these assignments (Fig. 1).

**Figure 2.**
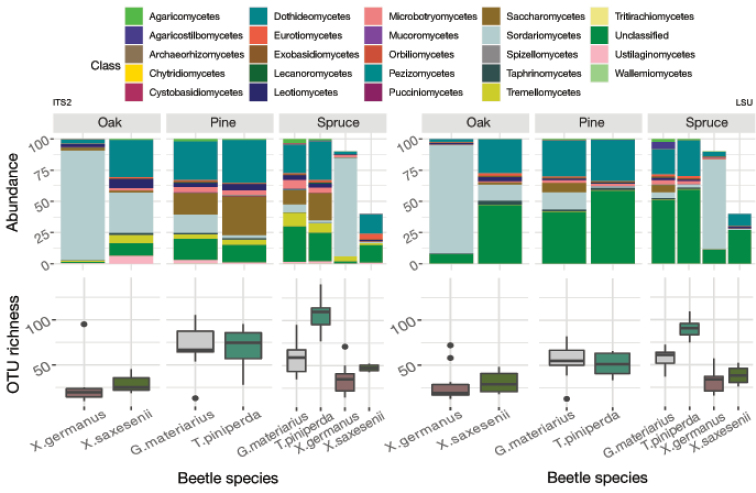
Top panel: The proportion of OTUs identified as members of a fungal Class determined by the ITS2 and LSU D1-D2 regions. For the spruce forest, only nine *X.germanus* and four *X.saxesenii* specimens were retained after rarefaction. Lower panel: The number of fungal OTUs per beetle specimen, separate for each beetle species and forest type, for ITS2 and LSU.

Libraries from 73 beetle specimens remained after rarefaction and harboured a total of 1180 OTUs for ITS2 and 553 OTUs for LSU D1-D2. Using taxonomic classifiers, OTUs were assigned to 24 classes, 66 orders, 129 families and 369 genera. Identification at class level revealed the presence of 23 classes for ITS2 and 17 classes for LSU. The dominant classes were Dothideomycetes for ITS2 and Sordariomycetes for LSU D1-D2 (Fig. 2, Suppl. material 6: Table S2). ITS2 produced twice as many identified OTUs compared to LSU D1-D2, and in the classes Leotiomycetes and Tremellomycetes more than five times as many, due to the greater total number of OTUs and the higher proportion being fully identified. ITS2 metabarcoding also detected seven fungal classes not retrieved with the LSU D1-D2 primers (Archaeorhizomycetes, Chytridiomycetes, Mucoromycetes, Orbiliomycetes, Spizellomycetes, Tritirachiomycetes and Ustilaginomycetes), while LSU D1-D2 metabarcoding recovered only one class not obtained with the ITS2 primers (Atractiellomycetes). Only for the Sordariomycetes and Agaricomycetes the proportion of OTUs detected with LSU D1-D2 was higher than with the ITS2 marker.

### ﻿Comparison of the ITS and LSU markers in ecological analyses

Fungal communities obtained with either marker were compared with regard to total richness and differentiation across beetle species and forest type. For both markers, species accumulation curves displayed a similar shape, despite the roughly twice higher OTU number in ITS2, with a slow increase and not reaching a plateau, although LSU D1-D2 generally showed a more pronounced ‘shoulder’ indicating a fraction of OTUs that is encountered commonly in multiple samples. Across the different forest types, species accumulation in oak forest clearly lagged pine and spruce forests (fewer total species, slower accumulation) in both markers (Suppl. material 2: Fig. S2).

**Figure 3.**
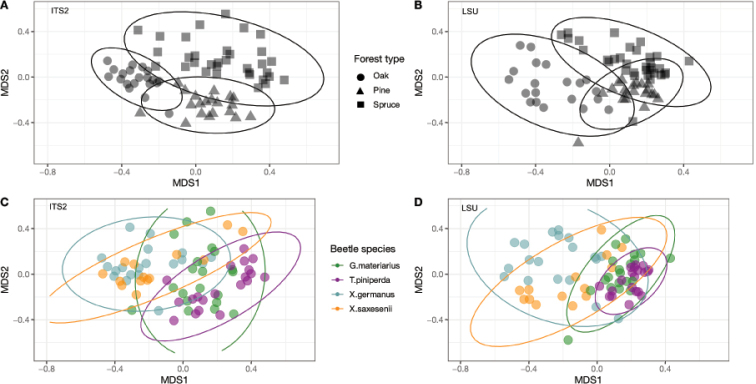
NMDS ordination plot of all specimens sampled with ITS2 and LSU D1-D2, based on the fungal community composition of the individual beetles. Shapes represent forest types and colours represent beetle species. Stress for this graph fell within acceptable ranges (<0.2).

Richness in a single-beetle extract ranged from 9 to 140 fungal OTUs (average 56 ± 32.34) in ITS2 and from 11 to 109 fungal OTUs (average 48 ± 24.27) in LSU D1-D2 (Fig. 2). Despite some scatter among individual beetles, the number of OTUs per sample differed in a characteristic way between beetle species and forest types, and these differences were closely correlated in ITS2 and LSU D1-D2, indicating that both markers detected a similar set of fungal species (beyond the classes unique to each marker, which only make a small contribution to overall species richness and relative abundances). This correlation was also evident at specimen level in the two outliers in each of the libraries corresponding to the same beetle individual. The variation in species richness explained by forest type and beetle species was broadly similar in ITS2 and LSU D1-D2 derived fungal communities (Table 2), although the LSU data attributed a greater proportion of the variation to the forest type alone (27.47% compared to 18.75% from ITS2), while the reverse was true for ITS2. Community composition analysed with both markers had around 8% of the variation explained by the interaction of beetle species and forest types. NMDS plots on the OTU composition revealed a very similar pattern of community separation of the three forest types in ITS2 and LSU (Fig. 3).

**Table 2.** Correlation of species richness with beetle species and forest type. The table shows the result of a GLM analysis showing the percentage of explained variance for each predictor with the F parameter and significance level.

Factor	Explained variance		*F_x,y_*		*p*	
	ITS2	LSU	ITS2	LSU	ITS2	LSU
**Beetle + forest type**	8.46%	7.58%	*F*_6, 65_ = 2.809	*F*_6, 65_ = 3.521	<0.1 *	<0.05 *
**Beetle**	27.07%	18.25%	*F*_3, 69_ = 30.265	*F*_3, 69_ = 29.888	<0.001 ***	<0.001 ***
**Forest type**	18.75%	27.47%	*F*_2, 67_ = 6.772	*F*_2, 67_ = 5.236	<0.01 **	<0.01 **
**Unexplained**	45.72%	46.68 %				

**Figure 4.**
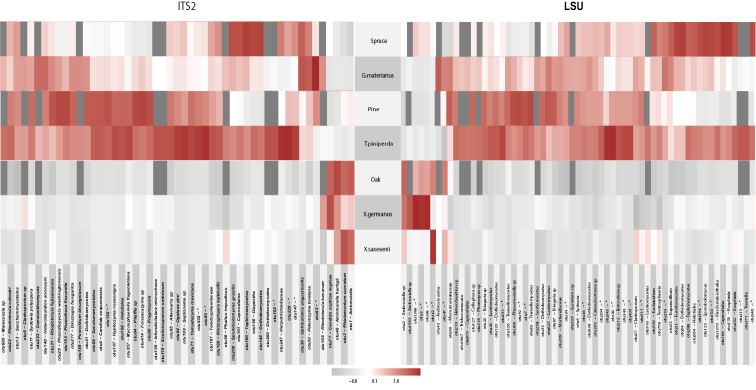
Heatmap using Pearson’s correlation coefficient between the OTUs generated from the ITS2 and LSU D1-D2 metabarcodes and the analysed beetle species and forest types. Rectangles indicate the strength of association between an OTU and beetle/forest (strongly negative, grey, to strongly positive, red). Fungal OTUs (on the horizontal axis) were classified to genus or species level where possible; they are shown in random order and cannot be linked taxonomically between both markers.

The *indval* function revealed significant levels of association with the tree species and or the beetle species for 50 and 60 OTUs, respectively, from the ITS2 and LSU D1-D2 regions. Many OTUs showed positive associations with pine and spruce, but much fewer with oak. Regarding the associations with beetle species, many OTUs had positive associations with *T.piniperda*, and to some extent with *G.materiarius*, whereas positive associations with *Xs.germanus* and *Xb.saxesenii* were limited to a small number of oak associated OTUs. Most other associations in these species were negative; e.g., the pine/spruce associated OTUs were absent, despite the fact that both beetle species were also sampled from spruce. General patterns of OTU associations and non-associations were similar for the two xyleborine species, and they were quite similar to those associated with oak. In contrast, association patterns in *T.piniperda* and *G.materiarius* were similar to pine and spruce (Fig. 4). The similarity in these association patterns differed only slightly between the ITS2 and LSU-based OTUs (Fig. 4), even though the OTUs themselves could not be linked up between the two markers, as they mostly were not identified to species level, or the identifications did not overlap between the two marker sets.

### ﻿OTU identifications across markers using phylogenetics

A phylogenetic approach was used to associate ITS-based and LSU-based OTUs with each other, focusing on the class Sordariomycetes that includes the Ophiostomatales of important tree pathogens for which ITS efficiency has been questioned (Skelton et al. 2019; Hulcr et al. 2020). OTUs were clustered at minimum similarity thresholds of 97, 98 and 99%, which resulted in between 120–150 OTUs for ITS2 and 80–120 OTUs for LSU D1-D2 classified as Sordariomycetes using the RDP classifier at > 80% confidence (Table 3). The most similar values for the number of OTUs were obtained at 98% and 99% thresholds for ITS2 and LSU D1-D2 (Table 3). As each species should produce one ITS and one LSU sequence, we used these as the preferred threshold values in further analyses. These conditions were used because they generated a similar number of OTUs for each marker (Table 3), and thus potentially represent a similar set of species.

**Table 3.** Sequence numbers and phylogenetic dispersion in SordariomycetesOTUs under different threshold values. The table presents the Net relatedness index (NRI), nearest taxon index (NTI), and the number of OTUs recovered for ITS2 and LSU D1-D2. “Mixed” refers to a clustering threshold of 99% for LSU D1-D2 and 98% for ITS2. Reference sequences were included when building the trees used, though pruned (leaving only OTUs in the tree) for the above calculations. Positive NRI and NTI scores indicate phylogenetic clustering of either ITS and LSU sequences (indicating different species sets were sequenced), negative values indicate phylogenetic over-dispersions of ITS and LSU with respect to each other (indicating the same species was sequenced for the two markers).

	ITS2					LSU		
	97%	98%	99%	Mixed	97%	98%	99%	Mixed
**NRI**	-0,111	-0,805	1,497	-0,122	1,328	0,697	-2,55	-0,653
**NTI**	-1,212	-3,81	-2,386	-2,277	1,367	0,882	-0,183	-1,343
**OTU count**	138	144	158	144	80	102	150	150

OTU sequences from both markers were included in a phylogenetic analysis together with publicly available full-length sequences covering the full or most of the rRNA cluster, including the ITS2 and LSU D1-D2 regions, with the SSU gene also present in most accessions. These sequences served as a scaffold to represent the major orders of Sordariomycetes (full list of accessions in Suppl. material 5: Table S1), to which the OTU sequences were added. ML trees for the combined three-region reference alignment and OTUs from metabarcoding resolved relationships at the base of the tree similarly to those found in the literature (Zhang et al. 2006; Hongsanan et al. 2017) (Suppl. material 3: Fig. S3). All orders were monophyletic, given the taxonomic assignment of the reference sequences in their Genbank accessions. The positions of OTUs on this tree were then used to provide a taxonomic assignment at the level of orders. This was achieved by determining the node representing the hypothetical ancestor of all reference sequences representing an order (based on their Genbank taxonomy), and OTUs descended from this ancestor were assigned to the same order. OTUs placed on branches outside of these clades were considered ‘unassigned’. By using this approach, 254 of the 294 OTUs were placed into clades defined by the reference sequences, thus determining their identity at order level. This number compared to 212 OTUs classified by RDP, 150 OTUs by IDTAXA (141) and 31 OTUs by Protax-fungi (ITS only). Out of these, 8, 9 and 3 OTUs were misclassified by the three classifiers, respectively. The few cases of disagreement of the phylogenetic analysis with the classifiers affected mainly OTUs that showed discrepancies of assignments between the classifiers.

OTUs obtained from ITS2 and LSU D1-D2 were widely distributed on this tree, and across most orders, both types of sequences were interleaved, showing that overall community diversity at the order level could equally be inferred using either region (Suppl. material 2: Fig. S2). Order-level subsets of trees for these orders showed the placement of ITS2 and LSU sequence fragments relative to the reference set (Fig. 6A, B). If both sequences are derived from the same genomic template in the metabarcoding amplification they were expected to be represented by one OTU representative sequence for each marker, and these sequences to fall in proximity on the tree, taking the same phylogenetic position relative to the nearest full-length reference sequence (Fig. 6, species D). We found 15 instances where one ITS and one LSU barcode were in close proximity together with a reference sequence (84 reference species in total), potentially representing the same species. In an additional six instances, one or both barcodes formed a cluster on zero-length branches when matched to full-length rRNA reference sequences, i.e., representing an exact match to an existing database entry, but missing the other type of barcodes.

Closed-reference clustering against the reference dataset to each order within Sordariomycetes by the RDP classifier revealed species-level matches for both ITS2 and LSU sequences (Fig. 7A). Notably, four species had matches to both markers, i.e., the same species were amplified. In addition, one ITS2 sequence produced a hit not reciprocated in LSU. Vice versa, LSU sequences produced hits to a minimum of eight additional species not seen in ITS, which was increased to 11 and 17 species under the higher 98 and 99% threshold values, respectively, as the trees became increasingly populated with the additional taxa from splitting of larger OTUs (Fig. 7B). Under these lower threshold values closely related sequences apparently were less affected by ‘lumping’, which obscured the true diversity in the sequencing mixture.

Where closely related reference sequences were missing, ITS2 and LSU sequences may be matched based on their phylogenetic proximity, but the ITS2 and LSU sequences obtained from a single genome may not appear as sister taxa because the gene sequences are non-overlapping and thus lack characters that could group them. We tested the degree to which ITS2 and LSU sequences interleave on the tree, by assessing phylogenetic clustering and dispersion with the NRI and NTI (Table 3). For ITS2, most values were negative, indicating over-dispersion relative to the LSU sequences as expected if both markers pick up the same or closely related species. The exception was for the 99% similarity value, which produced positive NRI (clustering) possibly from selective over-splitting of OTUs that was not matched in the less variable LSU sequences. For LSU there was a progression from positive (clustering) at 97% similarity to negative (indicating over-dispersion) at 99% similarity, which coincided with a near doubling in the number of OTUs (against only a small increase in the ITS2 data) (Table 3). This indicated that OTUs newly formed by splitting were not clustered on the tree, unlike the ITS2-derived OTUs, but instead were interleaved with the ITS2 sequences, indicating more complete representation of species already on the tree based on their ITS2 sequences. The ‘mixed’ threshold value of 98% for ITS2 and 99% for LSU presented slightly negative NRI/NTI values for both markers (Table 3).

**Figure 5.**
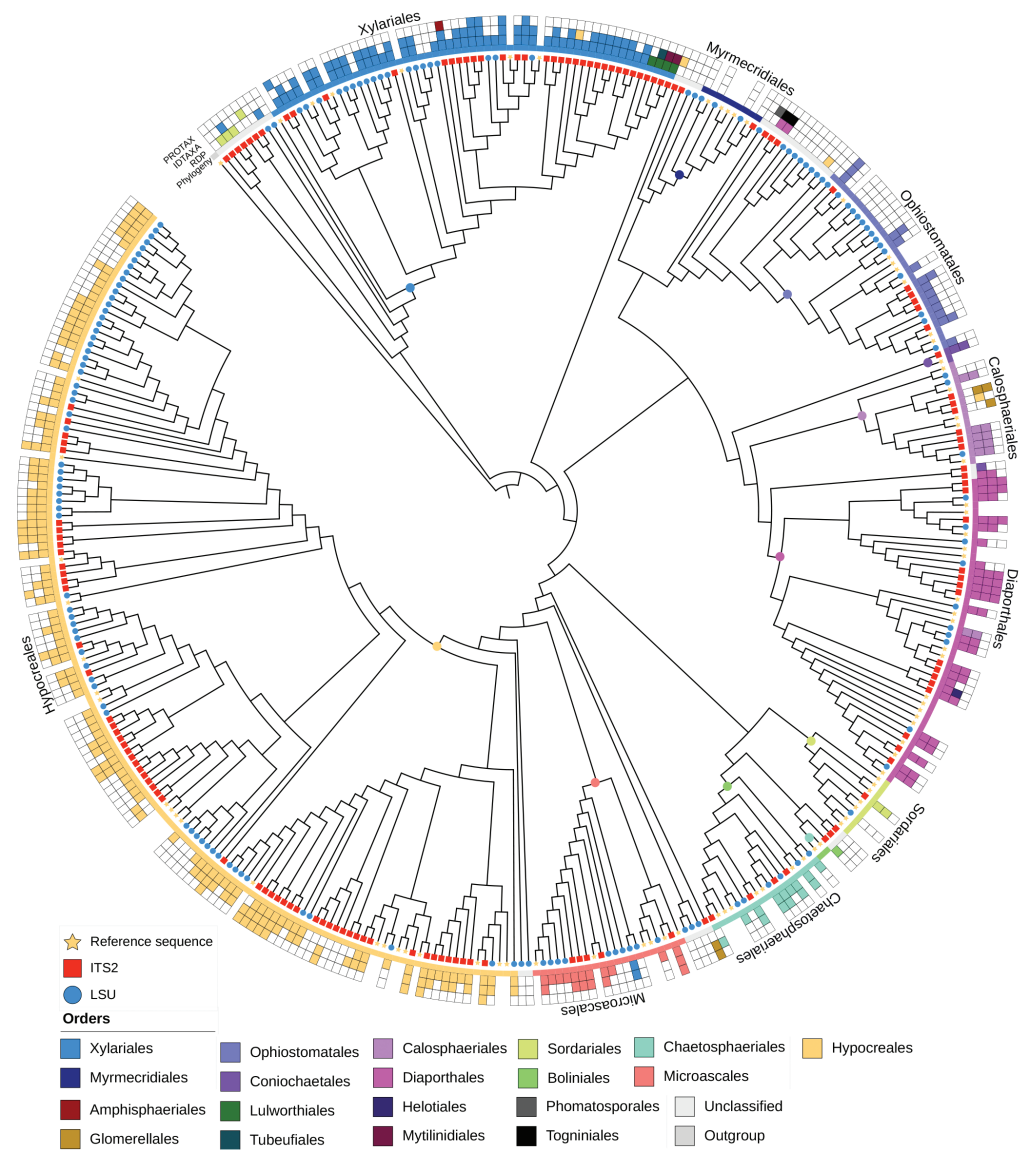
ML tree of Sordariomycetes constructed from the reference sequence alignments and OTUs for both markers (clustering thresholds: 98% ITS2, 99% LSU D1-D2). *Leotialubrica* (Leotiomycetes) was specified as the outgroup. The assignment of OTUs by each of the three classifiers (RDP, IDTAXA, Protax-fungi) is shown by coloured boxes. Terminals missing these boxes are the reference sequences. Coloured dots on the nodes of the tree indicate the hypothetical ancestor defining monophyletic groups corresponding to the various orders of Sordariomycetes. The extent of each order is indicated by the coloured inner ring. Note that the ancestor of an order is defined by the youngest node from which all reference sequences are descended; OTUs falling outside of the resulting clades appear as ‘unassigned’ by the phylogenetic analysis approach. The distribution of ITS2 (red squares) and LSU D1-D2 (blue bullets) relative to the reference set (yellow stars) on each of the tips of the tree. Note the limited presence of ITS sequences in the Ophiostomatales (in top right quadrant).

The detailed observations were confirmed by the global classification of OTUs at order level, which showed an increase in the proportion of identified OTUs with increasing threshold value for LSU, but not ITS2 (Fig. 8). Both markers produced broadly similar proportions of the four dominant orders, Xylariales, Ophiostomatales, Diaporthales and Hypocreales, but differed to various degrees in the assignment of the ‘small’ orders. It was also evident that OTU numbers in Ophiostomatales were comparatively lower in ITS2, as also suggested from the phylogenetic tree (Fig. 5). This is likely explained by the fact that the ITS2 forward primer binding site in this group differs from the consensus (Tedersoo et al. 2015b; also see Suppl. material 4: Fig. S4).

**Figure 6.**
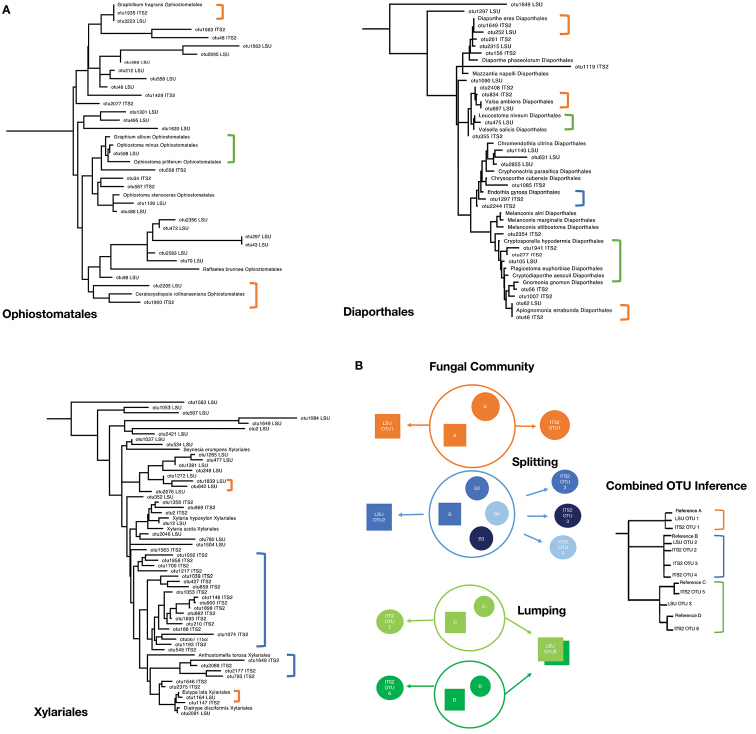
Order-level trees and splitting/lumping of OTUs at clustering **A** order-level ML trees with mixed OTU clustering thresholds (99% LSU D1-D2, 98% ITS2). Full tree in supplementary materials. *Leotialubrica* was used as the outgroup (not pictured). Brackets indicate reference taxa linked to an ITS2 and/or LSUOTU, with colours indicating potential splitting/lumping (blue, splitting; green, lumping; orange, 1:1) **B** diagram illustrating the effects of splitting and lumping of an OTU in the fungal community on the tree inference. Four hypothetical species (A to D) in a community are treated under uniform clustering thresholds for ITS2 and LSU. This may result in deviation from the 1:1 ratio of OTUs expected if each species in the community is represented equally by both markers (species A). Threshold values may be too high, resulting in splitting of species into multiples OTUs, which is likely to affect the more variable ITS2 region (species B) or may be too low, resulting in lumping of multiple species into a single OTU, likely to affect the conservative LSU region (species C and D).

**Figure 7.**
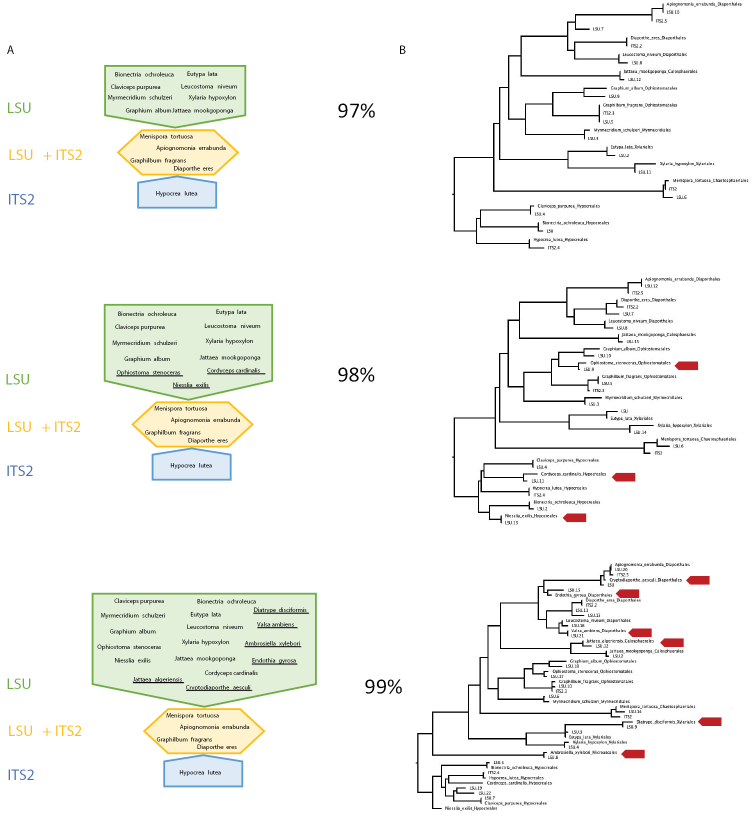
Closed reference clustering of OTUs and phylogenetic trees at different thresholds **A** results from the closed reference clustering of OTUs at each clustering threshold against composite LSU/ITS2 reference sequences. LSU matches in green, ITS2 matches in blue, linked matches (for which both an ITS2 and LSUOTU were matched to a reference sequence of the same species) in yellow. Underlined taxa indicate new matches at each clustering threshold **B** phylogenetic tree of LSUOTUs under increasingly stringent clustering thresholds, with arrows marking newly added taxa as threshold values are increased.

**Figure 8.**
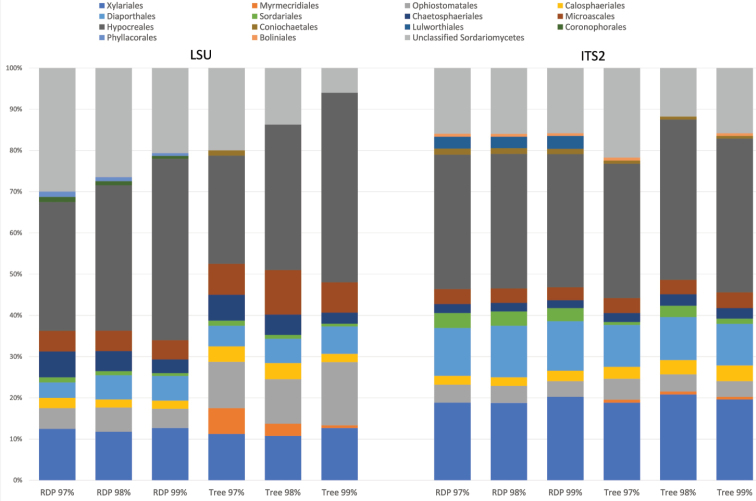
Proportion of OTUs assigned to each Order from metabarcoding with LSU (left panel) and ITS (right panel) markers based on the RDP classifier and the phylogenetic tree, under increasing threshold values.

## ﻿Discussion

Metabarcoding has revolutionised the study of fungal communities, revealing the huge proportion of hitherto unobserved species, including the unexpectedly high diversity of fungi associated with bark beetles (Miller et al. 2016; Hulcr et al. 2020; Větrovský et al. 2020). However, these inferences are based on short sequences and lack the biological information of conventional studies using fungal cultures. Independent corroboration of species limits is needed, and principally can be achieved by using multiple markers that each define the same entities (e.g. DeSalle et al. 2005). The test of phylogenetic congruence in metabarcoding data is complicated because the amplicons come from complex mixtures of species, which does not allow to establish genetic linkage (phasing) across the two markers, despite the proximity of the ITS2 and LSU D1-D2 regions in the genome. Instead, an indirect approach had to be used that identifies the amplicons of ITS2 and LSU separately relative to full-length reference sequences comprising the entire rRNA cistron.

We assessed the congruence of signal from ITS and LSU metabarcoding for the characterisation of fungal communities, by (1) comparing the ecological associations at various taxonomic levels as established with either marker (for the entire fungal set), and (2) testing the species-level correspondence of OTUs from both markers based on their phylogenetic positions (for the class Sordariomycetes only). As we showed in both cases, OTU identification is challenging and depends on the available reference databases, as well as the specific strategy for linking the metabarcode sequences into the taxonomic system. Taxonomic classifiers are now widely used and are becoming increasingly sophisticated. However, placement was possible mostly to higher taxonomic levels only (Fig. 1), in line with existing studies (Richardson et al. 2017). Just a small proportion of reads could be identified to genus or species, usually only with one of the three classifiers, while the LSU marker is not even annotated to species level in the RDP fungal training set. These difficulties in identifying species compromised the comparison of community composition obtained with either marker, as virtually none of the OTUs encountered in each set were labelled with the same Linnaean binomial (Suppl. material 3: Fig. S3).

In contrast, simple counts of OTU numbers (a proxy of species richness) produced a good correlation between both markers in several key parameters describing the community composition. First, the numbers of OTUs and the higher-level composition of fungal communities obtained from each treatment (beetle species, forest type) assessed with the ITS2 and LSU D1-D2 data closely mirror each other (Fig. 2). This also holds for the composition of orders within the class Sordariomycetes (Fig. 8). Equally, the proportions of explained OTU diversity by beetle species, forest type and beetle × forest interactions were remarkably similar between both markers, even if the absolute number of OTUs was much lower in LSU D1-D2 (Table 2). For both markers, communities from different forest types and beetle species occupy similar portions of the multivariate space (Fig. 3). Finally, the broad patterns of individual OTU associations in the *indval* analysis show similar affinities with the beetle species and tree type (Fig. 4), even if the correspondences of species between ITS2 and LSU D1-D2 datasets could not be determined. All these findings point to a high level of congruence between both markers and provide justification for the widely used approach of fungal community analysis using metabarcoding with either marker, based on higher level classification and read abundances. The utility of read abundance in these analyses is particularly remarkable given the frequently raised concern about skew in the number of reads in the PCR (Bálint et al. 2016; Krehenwinkel et al. 2017). Thus, even a single metabarcode marker can safely represent the broad ecological trends determining fungal communities, as previously found in studies addressing a wide range of ecological questions (Tedersoo et al. 2015b; George et al. 2019; Nilsson et al. 2019; Furneaux et al. 2021).

Yet, the difficulty of linking these metabarcoding sequences across multiple markers leaves some uncertainty about the biological relevance of the community data, which still may represent different species within the major taxonomic groups recovered by either ITS2 and LSU D1-D2, as already suggested for the Ophiostomatales (Hulcr et al. 2020). Thus, ultimately, the metabarcoding approach may fall short of linking any particular fungal species to a beetle, unlike the conventional approaches of culturing particular isolates that reveal the specific symbioses. Phylogenetic analysis of individual sequences can improve the precision of identification with both markers individually and relative to each other, beyond the assignment to a broad taxonomic group, and thus link the corresponding reads representing a given species from either marker (Fig. 6D).

As illustrated for the Sordariomycetes, we found that OTU assignments obtained by taxonomic classifiers are broadly in agreement with the phylogenetic analysis. The backbone of the phylogenetic tree from the full-length rRNA reference sequences recovered each order of the Sordariomycetes as monophyletic (Fig. 5, Suppl. material 3: Fig. S3). OTUs placed on this tree can then be scored for membership in clades defined by the reference sequences. The RDP classifications and tree-based assignments were largely in agreement regarding the species composition at order-level, although generally the trees assigned a greater proportion of OTUs, reaching nearly 95%. When placed on the tree, the order level assignments were consistent with the identifications obtained by the classifiers, and disagreements mainly affected cases where only one of the classifiers disagreed or the alternative identifications differed between classifiers (Fig. 5). This observation suggests low confidence in the conflicting identifications, as also indicated by the average confidence scores from the RDP classifier that varied between orders, with LSU assignments having low confidence overall (see Suppl. material 7: Table S3). Many OTUs were not identified beyond the class level by the classifier, despite clear placement in the tree. The order Myrmecridiales was missing entirely from the classifier results, despite the presence of several OTUs placed clearly within the order and OTUs matching *Myrmecridiumschulzeri* found in the closed-reference clustering at all three thresholds (Fig. 7A, B). The comparison with formal phylogenetic analyses thus highlights the limitations of classifiers that are dependent on reference databases and probabilistic *k-mer* matching, given the limited sequence length of metabarcoding reads (Wang et al. 2007; Porras-Alfaro et al. 2014; Bacci et al. 2015; Xue et al. 2019).

Second, we used the phylogenetic analysis to determine if both markers reveal the same species-level entities. Under ideal circumstances, each species is represented by exactly one sequence each of LSU and ITS, and these two sequences from both markers find themselves in the same position of the tree. As both markers are non-overlapping, they only can be placed relative to full-length reference sequences rather than to each other, and therefore if 1:1 represented for each species, sequences of ITS and LSU should be uniformly interleaved on the tree (Fig. 6D). However, if similarity thresholds are too low (incorrectly lumping of species) or too high (splitting of species) in one or both of these loci, deviations from the uniform distribution occur. Overall, the increase of the similarity threshold had a greater impact on the LSU D1-D2 than ITS2, almost doubling in numbers of recognised OTUs versus a small increase only (Table 3), and parity of OTUs in both markers was greatest at a ‘mixed’ threshold of 99% for LSU D1-D2 and 98% for ITS2. While simplistic, the logic of this analysis is straightforward and the results could be improved with greater density of reference sequences. Using the NRI/NTI framework under these OTU thresholds (Table 3), ‘communities’ of LSU and ITS2 sequences show over-dispersion, as expected for the 1:1 correspondence of each marker. The 99% threshold for LSU is also supported by the greater matches in the closed-reference clustering (Fig. 7). Because of the similarities in OTU counts and because of the NRI/NTI values indicating moderate levels of over-dispersion, we consider the 98/99% threshold mixed strategy as the best estimate of the OTU diversity in each marker. Thus, proximity on the tree is taken to indicate that the respective ITS2 and LSU sequences are derived from the same genomic template, or at least from closely related strains present in a community. Frequently this was corroborated by the fact that these closely related ITS2 and LSU sequences were obtained from the same specimen sample (not shown).

There are uncertainties associated with this inference. Across fungal species, intraspecific ITS variability varies considerably, highlighting the challenges and inevitable shortcomings resulting from the selection of a uniform OTU clustering threshold (Nilsson et al. 2008). For example, while a 97% clustering threshold is generally accepted and widely used in environmental sequencing studies (Kõljalg et al. 2013; Tedersoo et al. 2015b), other studies from sequencing of well-defined strains from culture collections have suggested a much higher optimal threshold of > 99.6% similarity (Vu et al. 2019). However, with the use of long-read technology the full extent of intraspecific and intragenomic variability is becoming evident. For example, in *Xylariahypoxylon* more than a dozen copies of the rRNA cistron were detected in a single genome, with ITS sequence divergences ranging from 96.9–99.8% (Stadler et al. 2020). Although intra-genomic variation in other species of Xylariales was lower, this case demonstrates the difficulty of splitting vs. lumping in the analysis of both markers. Thus, the higher number of ITS2 OTUs in Xylariales compared to LSUOTUs from the same communities (Fig. 6) may be the result of over-splitting of distantly related copies of ITS2 present in a single genome (Nilsson et al. 2008; Stadler et al. 2020). Yet, even if the clustering is not a correct reflection of intra-genomic and intra-specific variation, the placement of sequences representing the OTUs can link close relatives across different markers. For greatest success, densely sampled reference sequences spanning both markers are required, but as shown for the Sordariomycetes, even an incomplete set can provide the scaffold for placing non-overlapping sequences, and in several instances the idealised placement of OTUs and reference sequences was found, in some cases across all clustering thresholds (Fig. 5), while uncertainties remain where reference sequences are distant. Matching of ITS2 and LSU sequences was even possible in the Ophiostomatales, despite the deviation in the ITS2 primer binding site in this group (Tedersoo et al. 2015b; Suppl. Fig. S4), as the bias against the amplification presumably is overcome by permissive PCR conditions, and similar effects can be expected in other groups where such sequence variation may exist, e.g. in a clade of Hypocreales composed entirely of LSU sequences, although this is an exception in the taxonomically broad set of fungal lineages used here.

## ﻿Conclusion

We addressed the problem of marker choice in fungal metabarcoding for the study of biodiversity patterns and taxonomic identifications. Community-level diversity metrics showed high consistency of results from metabarcoding with both the ITS2 and LSU D1-D2 regions, using OTU clustering under the widely used 97% threshold level. However, when identified with standard taxonomic classifiers, great discrepancies in the taxonomic composition at species level were evident between both markers. We attempted to reconcile the two distinct ‘images’ of the community using a phylogenetic approach that incorporates barcodes from both regions into a single phylogeny generated from reference sequences covering the full rRNA cistron. We find that the ITS2 and LSU D1-D2 metabarcodes are broadly interleaved in these trees, linking individual sequences across markers. This analysis also was used to select threshold values for clustering in each marker, recommending a mixed strategy of 98% similarity for ITS2 and 99% similarity for LSU D1-D2. Phylogenetic approaches which, unlike taxonomic classifiers, do not rely on sequence similarities with marker-specific reference sets, can link barcodes from different regions and provide greater precision of taxonomic placement. In addition, the approach provides a means to evaluate threshold values for clustering; despite the general tendency for the use of denoised ‘exact sequence reads’ (ASVs; Callahan et al. 2017), metabarcoding with ITS and LSU markers may continue to require OTU clustering due to the problem of intra-genomic variation in these tandemly repeated markers.

## Supplementary materials

Supplementary material 1 Figure S1. Length distribution of the ITS (grey) and LSU (orange) OTUs Data type Eps file. Explanation note The average length for each amplified region is indicated with a dashed line. https://binary.pensoft.net/file/655182This dataset is made available under the Open Database License (http://opendatacommons.org/licenses/odbl/1.0/). The Open Database License (ODbL) is a license agreement intended to allow users to freely share, modify, and use this Dataset while maintaining this same freedom for others, provided that the original source and author(s) are credited.Angelina Ceballos-Escalera, John Richards, Maria Belen Arias, Daegan J. G. Inward, Alfried P. Vogler

Supplementary material 2 Figure S2. Species accumulation curves of the OTUs generated from the ITS (panel right) and LSU (panel left) metabarcodes Data type Eps file. Explanation note Colours show the different forest types in which beetles were trapped: oak (red), spruce (blue) or pine (green). https://binary.pensoft.net/file/655183This dataset is made available under the Open Database License (http://opendatacommons.org/licenses/odbl/1.0/). The Open Database License (ODbL) is a license agreement intended to allow users to freely share, modify, and use this Dataset while maintaining this same freedom for others, provided that the original source and author(s) are credited.Angelina Ceballos-Escalera, John Richards, Maria Belen Arias, Daegan J. G. Inward, Alfried P. Vogler

Supplementary material 3 Figure S3. Maximum-likelihood tree constructed in IQ-Tree2 based on three-gene (LSU D1-D2, SSU, ITS2) reference sequence alignments and OTUs for both markers (clustering thresholds: 99% LSU D1-D2 and 98% ITS2) Data type Pdf file. Explanation note *Leotialubrica* (Leotiomycetes) used as the outgroup. Node values indicate ultrafast bootstrap approximation support (n = 1000). https://binary.pensoft.net/file/655184This dataset is made available under the Open Database License (http://opendatacommons.org/licenses/odbl/1.0/). The Open Database License (ODbL) is a license agreement intended to allow users to freely share, modify, and use this Dataset while maintaining this same freedom for others, provided that the original source and author(s) are credited.Angelina Ceballos-Escalera, John Richards, Maria Belen Arias, Daegan J. G. Inward, Alfried P. Vogler

Supplementary material 4 Figure S4. Binding site of ITS86 primer showing mismatched base pairs in Ophiostomatales Data type Eps file. Explanation note Degenerate primer suggestion with variable base pairs in bold. While all other reference sequences were consistent with the non-Ophiostomatales sequences, only several are shown for clarity. https://binary.pensoft.net/file/655185This dataset is made available under the Open Database License (http://opendatacommons.org/licenses/odbl/1.0/). The Open Database License (ODbL) is a license agreement intended to allow users to freely share, modify, and use this Dataset while maintaining this same freedom for others, provided that the original source and author(s) are credited.Angelina Ceballos-Escalera, John Richards, Maria Belen Arias, Daegan J. G. Inward, Alfried P. Vogler

Supplementary material 5 Table S1. Accession numbers corresponding with the reference sequences used to build the phylogenetic trees Data type Xlsx file. https://binary.pensoft.net/file/655186This dataset is made available under the Open Database License (http://opendatacommons.org/licenses/odbl/1.0/). The Open Database License (ODbL) is a license agreement intended to allow users to freely share, modify, and use this Dataset while maintaining this same freedom for others, provided that the original source and author(s) are credited.Angelina Ceballos-Escalera, John Richards, Maria Belen Arias, Daegan J. G. Inward, Alfried P. Vogler

Supplementary material 6 Table S2. Class level identification of OTUs showing the number of OTUs produced with ITS2 and LSU and the proportion of the total OTU set on the rarefied data Data type Xlsx file. Explanation note OTUs classified at species level but not correctly classified at class level were considered as “Misclassified”. https://binary.pensoft.net/file/655187This dataset is made available under the Open Database License (http://opendatacommons.org/licenses/odbl/1.0/). The Open Database License (ODbL) is a license agreement intended to allow users to freely share, modify, and use this Dataset while maintaining this same freedom for others, provided that the original source and author(s) are credited.Angelina Ceballos-Escalera, John Richards, Maria Belen Arias, Daegan J. G. Inward, Alfried P. Vogler

Supplementary material 7 Table S3. Number of OTUs assigned to each order based on RDP Bayesian classifier Data type Xlsx file. Explanation note Average confidence scores were calculated over all order-level assignments, though only classifications with > 50% confidence were taxonomised, all others were kept as “unclassified Sordariomycetes”. https://binary.pensoft.net/file/655188This dataset is made available under the Open Database License (http://opendatacommons.org/licenses/odbl/1.0/). The Open Database License (ODbL) is a license agreement intended to allow users to freely share, modify, and use this Dataset while maintaining this same freedom for others, provided that the original source and author(s) are credited.Angelina Ceballos-Escalera, John Richards, Maria Belen Arias, Daegan J. G. Inward, Alfried P. Vogler
