# Supplementary material for: ﻿Metabarcoding of insect-associated fungal communities: a comparison of internal transcribed spacer (ITS) and large-subunit (LSU) rRNA markers
Source: MycoKeys. 2022 Mar 8;88:1–33. doi: 10.3897/mycokeys.88.77106 (PMC8924126; doi:10.3897/mycokeys.88.77106)

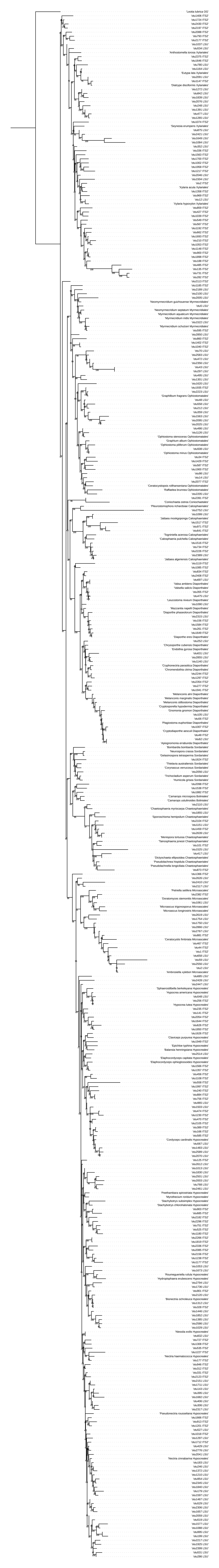

Supplement: Supplementary material 3 — Figure S3. Maximum-likelihood tree constructed in IQ-Tree2 based on three-gene (LSU D1-D2, SSU, ITS2) reference sequence alignments and OTUs for both markers (clustering thresholds: 99% LSU D1-D2 and 98% ITS2) [file mycokeys-88-001-s003.pdf]
